# Letter to the Editor Regarding “Differing Impacts of Cardiac Implantable Electronic Device Leads on Tricuspid Regurgitation”

**DOI:** 10.1002/joa3.70162

**Published:** 2025-08-06

**Authors:** Syed Sardar Shah, Ziad Khan, Attiq Ur Rehman, Shah Nawaz, Saleem Jan

**Affiliations:** ^1^ Saidu Medical College Mingora Pakistan

**Keywords:** cardiac implantable electronic devices, defibrillator, pacemaker, tricuspid regurgitation

Dear Editor,

I read with great interest the recent article by Leon, S. A., Austin, M. A., Parikh, C., Ahmad, D., Tchantchaleishvili, V., and Pavri, B. B. titled “Differing impacts of cardiac implantable electronic device leads on tricuspid regurgitation” (*Journal of Arrhythmia* 2025;41:e70133). Their insights into how different types and positions of CIED leads can affect the heart are truly valuable. I appreciate that they acknowledged the study's limitations, such as relying on a single operator, the retrospective design, and the small number of participants [1]. However, I believe there are a few more points worth considering to fully understand and interpret their findings. I hope that future studies can address these concerns, perhaps through prospective, multicenter designs with standardized imaging and detailed device information, which would really help clarify the actual impact of CIED leads on how the tricuspid valve functions. I commend the authors for their significant contribution to this field and encourage further exploration of this important clinical topic.

Here are some additional thoughts:

## 1 Confounding by Baseline Patient Characteristics

The ICD group had significantly lower baseline left ventricular ejection fraction (LVEF) and higher rates of right ventricular (RV) dilation and dysfunction compared to the pacemaker groups. These pre‐existing structural abnormalities are well‐known contributors to functional tricuspid regurgitation (TR) and may complicate the relationship between device lead type and TR progression. Without multivariable adjustment, the worsening TR observed may reflect disease severity rather than lead characteristics [2, 3].

## 2 Crude Categorization of Device Types

The broad grouping of devices into ICD, RV‐PM, and His‐PM overlooks differences in lead design, number of coils, stiffness, and positioning. Different lead models or materials may exert varied mechanical forces on the tricuspid apparatus. A more detailed device‐level analysis would provide much clearer conclusions [4].

## 3 Non‐Standardized Echocardiographic Assessment

TR severity was classified based on echocardiographic reports using qualitative terms (mild, moderate, severe), which are inherently subjective. Current guidelines recommend more precise multiparametric quantification (e.g., vena contracta width, PISA method), which reduces interobserver variability and misclassification bias [5].

## 4 Timing Heterogeneity in Follow‐Up Imaging

The interval between device implantation and follow‐up echocardiography varied significantly from 11 to 47 months. This inconsistency introduces a potential confounding factor from interim clinical events, such as heart failure progression or medication changes, which may influence TR severity independently of the device [6].

## Conflicts of Interest

The authors declare no conflicts of interest.

## Linked Articles

Leon SA, Austin M, Nasher N et al., “Differing Impacts of Cardiac Implantable Electronic Device Leads on Tricuspid Regurgitation,” *Journal of Arrhythmia*, https://doi.org/10.1002/joa3.70133.
